# The Scope of the Problem

**Published:** 2004

**Keywords:** underage drinking, adolescent, survey, AODU (alcohol and other drug use) pattern, binge drinking, AOD (alcohol and other drug) induced risk, risk and protective factors, alcohol and other drug related (AODR) consequences, AODR injury, epidemiology, special populations, children of alcoholics, undergraduate student, military, ethnic group, gender differences

## Abstract

Alcohol is the drug of choice among youth, often with devastating consequences. Alcohol is a leading contributor to injury death, the main cause of death for people under age 21. Drinking early in life also is associated with an increased risk of developing an alcohol use disorder at some time during the life span. Data consistently indicate that rates of drinking and alcohol-related problems are highest among White and American Indian or Alaska Native youth, followed by Hispanic youth, African Americans, and Asians. Prevalence rates of drinking for boys and girls are similar in the younger age groups; among older adolescents, however, more boys than girls engage in frequent and heavy drinking, and boys show higher rates of drinking problems. This article summarizes research on the epidemiology of youth drinking, including the consequences of youthful drinking, risk and protective factors and drinking trajectories, and information on special populations at particular risk for drinking-related problems.

## Overview

National surveys make it clear that alcohol drinking among youth is both widespread and harmful. Surveys provide data not only on the numbers of middle and high school students who drink but also on how they drink. The data show that when youth drink, they drink heavily in comparison with adults, consuming on average four to five drinks per occasion about five times a month, compared with two to three drinks per occasion about nine times a month for adults. Studies also find that drinking often begins at very young ages; a recent survey found that more than one-fourth of 14-year-olds reported drinking within the last year.

The negative consequences of under-age drinking include a range of physical, academic, and social problems. Perhaps most frightening, alcohol is the leading contributor to injury death, the main cause of death for people under age 21. However, alcohol also plays a powerful role in risky sexual behavior, including unwanted, unintended, and unprotected sexual activity, and sex with multiple partners. Alcohol is associated with academic failure and drug use. Over the longer term, data have shown that drinking early in life is associated with an increased risk of developing an alcohol use disorder at some time during the life span.

Although almost all U.S. youth grow up in a culture permeated by alcohol, they are not uniformly at risk for alcohol consumption or its consequences. Epidemiology provides clues to risk and protective factors associated with youth drinking, including family history and genetic vulnerability, comorbid conditions, sociodemographic characteristics, social stressors such as poverty and lack of social support, family characteristics, alcohol availability, temperament, and other individual factors. Epidemiology also provides a profile of how specific populations of young people differ in their drinking patterns. Drinking, including heavy drinking, is common and accepted among college students, with consequences affecting both those who do the drinking and those who do not. Rates of heavy drinking among 18- to 25-year-olds in the military are much higher than among civilians. There is considerable variation between Whites and other ethnic/racial minority youth with respect to drinking, but also significant variation within these populations. Research is needed to determine how national origin, tribal affiliation, acculturation, immigration status, and language all influence drinking patterns among youth.

## Epidemiology of Underage Drinking

Alcohol is the drug of choice among youth. Young people drink too much and at too early an age, thereby creating problems for themselves, for people around them, and for society as a whole. Hence, underage drinking is a leading public health problem in this country.

### Prevalence and Age of Initiation

Nationwide surveys, as well as studies in smaller populations, show that alcohol drinking is widespread among adolescents. For example, 2004 data from Monitoring the Future (MTF), an annual survey of U.S. youth, show that more than three-fourths of 12th graders, nearly two-thirds of 10th graders, and more than two in five 8th graders have consumed alcohol at some point in their lives (Monitoring the Future Web site). And when youth drink, they tend to drink heavily. Underage drinkers consume on average four to five drinks per occasion about five times a month (Substance Abuse and Mental Health Services Administration [SAMHSA] 2003). By comparison, adult drinkers ages 26 and older consume on average two to three drinks per occasion about nine times a month. A particularly worrisome aspect of underage drinking is the high prevalence of heavy episodic drinking, defined as drinking five or more drinks in a row in the past 2 weeks. MTF data show that 12 percent of 8th graders, 22 percent of 10th graders, and 28 percent of 12th graders engage in heavy episodic drinking (Johnston et al. 2004). It should come as no surprise, then, that about three-fifths of 12th graders, two-fifths of 10th graders, and one-fifth of 8th graders say they have been drunk (Monitoring the Future Web site). In fact, the highest prevalence of dependence is seen in people ages 18–24.

Studies also indicate that drinking often begins at very young ages. Data from recent surveys show that approximately 10 percent of 9- to 10-year-olds have already started drinking (Donovan et al. 2004), nearly a third of youth begin drinking before age 13 (Grunbaum et al. 2004), and more than one-fourth of 14-year-olds report drinking within the past year (SAMHSA 2003). Other researchers have documented that drinking becomes increasingly common through the teenage years (e.g., O’Malley et al. 1998). In addition, a number of studies have documented that the early onset of alcohol use (usually set at age 13 and younger) as well as the escalation of drinking in adolescence are both risk factors for the development of alcohol-related problems in adulthood (e.g., Gruber et al. 1996; Grant and Dawson 1998; Hawkins et al. 1997; Schulenberg et al. 1996*a*).

These findings clearly are cause for concern, as are recent data suggesting that the age of first use of alcohol is declining (SAMHSA, National Household Survey on Drug Abuse [NHSDA] for years prior to 2000). These data indicate that the average age of first use among young people of all ages was about 16 in 1999, compared with about 17 ½ in 1965 (SAMHSA 2003). Looking at underage drinkers only, 12- to 18-year-olds who report drinking report that they began doing so between 2 and 3 years earlier, when they were about 9 to 15, respectively (SAMHSA 2003). This is important because, as already noted, initiating alcohol consumption earlier in adolescence or in childhood is a marker for later problems, including heavier use of alcohol and other drugs during adolescence (e.g., Robins and Przybeck 1985; Hawkins et al. 1997) and meeting criteria for an alcohol dependence diagnosis in adulthood (Grant and Dawson 1998).

Most of what we know about under-age drinking derives from studies of youth ages 12 to 21. To address alcohol-related problems as developmental phenomena, we will need to understand more about what happens before age 12 with regard to alcohol consumption, alcohol awareness, and alcohol expectancies among children who have started to drink and among those who have not. A recent Medline search found a dearth of studies addressing drinking by younger children, and the few existing studies that turned up in this search were conducted among non-U.S. populations. Two national data sets, however, address alcohol use by children in sixth grade or below (typically age 12 and younger), albeit imperfectly and far from comprehensively. One is the Partnership Attitude Tracking Study (PATS), carried out for the Partnership for a Drug-Free America in 1993, and annually from 1995 through 1999. The other is the collection of PRIDE surveys carried out during the academic years 1997–1998 through 2001–2002. PATS data reveal a tripling of alcohol experience between fourth and sixth grade: 9.8 percent of fourth graders, 16.1 percent of fifth graders, and 29.4 percent of sixth graders report trying more than a sip of alcohol (Donovan et al. 2004). PRIDE data show similar rates of use in this population. Despite methodological problems with these data sets, PATS and PRIDE show that a nontrivial level of alcohol consumption occurs among a significant proportion of the 12-and-under population.

### Consequences

Underage drinking can result in a range of adverse short-term and long-term consequences, including:

- Academic problems
- Social problems
- Physical problems such as hangovers or medical illnesses
- Unwanted, unintended, and unprotected sexual activity
- Physical and sexual assault
- Memory problems
- Increased risk for suicide and homicide
- Alcohol-related car crashes and other unintentional injuries such as burns, falls, and drownings
- Death from alcohol poisoning
- Alterations in brain development that may have consequences reaching far beyond adolescence.

Alcohol is a leading contributor to injury death, the main cause of death for people under age 21. Annually, about 5,000 youth under age 21 die from alcohol-related injuries that involve underage drinking. This includes injuries sustained in motor vehicle crashes (about 1,900), homicides (about 1,600), and suicides (about 300), as well as unintentional injuries not related to motor vehicle crashes (National Highway Traffic Safety Administration [NHTSA] 2003; Centers for Disease Control and Prevention [CDC] 2004; Smith et al. 1999; Levy et al. 1999; Hingson and Kenkel 2004). Furthermore, the role of alcohol in both fatalities and injuries may be significantly underreported, in part because in many States, alcohol involvement in an injury relieves insurance providers of liability for medical expenses, so health care providers may not ask victims about, or report, alcohol use.

Numerous cases of alcohol poisoning, the result of the acute toxic effects of alcohol that can range from gastritis to severe gastrointestinal bleeding to respiratory arrest and death, have been reported in the news media. Although many of these tragedies occur on college campuses, especially striking was the recent report of two 11-year-old boys found dead of alcohol poisoning in a snowy field on the Flathead Indian Reservation in Montana, with blood alcohol concentration (BAC) levels of 0.20 percent and 0.50 percent. Although alcohol poisoning is by no means a major cause of death among youth, reports such as this underscore the tragic influence that hazardous drinking can wield over youth culture.

In the National Longitudinal Alcohol Epidemiologic Survey (NLAES) of people ages 18 and older in the United States, people who reported starting to drink before the age of 15 were four times more likely to also report meeting the criteria for dependence at some point in their lives (Grant and Dawson 1998). This survey also shows that children who drink at age 14 or younger are much more likely during their lifetimes to sustain unintentional injuries, to get into physical fights, and to become involved in motor vehicle crashes after drinking (Hingson et al. 2000, 2001, 2002).

Similarly, other survey data indicate that the younger children and adolescents are when they start to drink, the more likely they are to engage in behaviors that can harm themselves and others (Grunbaum et al. 2004). Those who start to drink before age 13, for example, are nine times more likely to binge^1^ drink frequently (five or more drinks on an occasion at least six times per month) as high school students than those who begin drinking later. And compared with nondrinkers, a greater proportion of frequent binge drinkers (nearly 1 million high school students nationwide) engaged in other risky behavior in the past 30 days (Grunbaum et al. 2004), including carrying a gun (22 percent vs. 3 percent), using marijuana (73 percent vs. 7 percent), using cocaine (26 percent vs. 0 percent), and having sex with six or more partners (31 percent vs. 4 percent). In addition, these youth were more likely than abstainers to earn grades that are mostly Ds or Fs in school (15 percent vs. 5 percent), be injured in a fight (13 percent vs. 2 percent), or be injured in a suicide attempt (9 percent vs. 1 percent). The extent to which alcohol use per se makes these other outcomes more likely is yet to be determined. However, the longitudinal evidence is very strong that the risk factors predicting earlier alcohol use also are strong predictors of virtually all of these other consequences (Biglan et al. 2004; Caspi et al. 1997).

### Risk Trajectories and Drinking Trajectories

Not only do youth begin drinking at different ages but their trajectories of risk also vary considerably, even before alcohol use has begun. Recent work following high-risk populations of children from preschool onward has shown major differences in the trajectories of externalizing and internalizing risk from preschool to early adolescence. These varied as a function of initial level of risk in early childhood, the child’s age, and the level of familial risk. Particularly for the externalizing trajectory, children who started out at very high levels of individual and familial risk became indistinguishable from those at lower levels during the early school years, but as these youth moved into early adolescence, the differences reemerged and became amplified, with the highest-risk children increasing the greatest amount (Zucker et al. 2003). Conversely, the externalizing behavior of children at the lowest level of initial risk who were exposed to the lowest level of familial risk changed the least, although even they increased in level of externalizing behavior as they moved into adolescence.

**Consequences of Underage Drinking: Mortality From Alcohol-Related Injuries**

Annually, about 5,000 people under age 21 die from alcohol-related injuries involving under-age drinking, including:

- Motor vehicle crashes – 1,900
- Homicides – 1,600
- Suicides – 300

SOURCE: National Highway Traffic Safety Administration 2003; Centers for Disease Control and Prevention 2004; Smith et al. 1999; Levy et al. 1999; Hingson and Kenkel 2004. All statistics are approximate.

Similarly, the drinking patterns and practices youth adopt as they grow into young adults—their drinking trajectories—also vary considerably once they start to drink. No single trajectory describes the course of alcohol use for all or even most young people. Research findings provide strong evidence for wide developmental variation in drinking patterns in the population. For example, Steinman and Schulenberg (2003) identified six common trajectories among early and middle adolescents: abstinence, rare use, high school onset, early but nonescalating use, early and gradually escalating use, and consistently high use. In another study, Schulenberg and colleagues (1996*b*) identified six trajectories of heavy drinking among young people ages 18 to 24: chronic heavy drinkers, decreased, increased, fling (i.e., low, high, low), rare, and never. In addition, alcohol abuse treatment (Chung et al. 2003) and other experiences may influence drinking trajectories. Studying the developmental trajectories of drinking behavior and how various risk and protective factors influence those trajectories is critical to understanding the complexity of underage drinking.

## Special Populations of Young People

### Children of Alcoholics

Children of alcoholics (COAs) are between 4 and 10 times as likely to become alcoholics themselves as children from families that have no adults with alcoholism (Russell 1990). COAs are at elevated risk for earlier onset of drinking (Donovan et al. 2004) and earlier progression into drinking problems (Grant and Dawson 1998). Some of the elevated risk is attributable to exposure and socialization effects found in alcoholic households, some to genetically transmitted differences in response to alcohol that make the drinking more pleasurable and/or less aversive, and some is attributable to elevated transmission of risky temperamental and behavioral traits that lead COAs, more than other youth, into increased contact with earlier-drinking and heavier-drinking peers.

From a public health standpoint, according to NLAES data (Grant 2000), approximately 9.7 million children age 17 or younger, or 15 percent of the child population in that age range, were living in households with one or more adults classified as having an alcohol abuse or dependence diagnosis during the past year. Approximately 70 percent of these children were biological, foster, adopted, or stepchildren. That is, 6.8 million children meet the formal definition of COA, although not all are exposed to the same level of risk for use, problem use, and alcohol use disorder (AUD). Given that these figures concern past-year exposure to at least one alcoholic adult, from the perspective of socialization risk, they only reflect acute exposure. Other data from NLAES provide estimates of the number of children living in a household with an adult who had abused or been dependent on alcohol at some point; the figure is 43 percent of the under-18 population, or somewhat less than half of all children. Given the size of this group, any approach to risk identification will be extremely complex.

A second important consideration is that COA status is heavily used as a proxy for “alcoholism risk” on the one hand and socialization risk on the other, but the COA designation more precisely is a proxy for multiple causal inputs, not all of which may be present in the individual case. Thus, being a COA implies elevated genetic risk, although the alcoholic genetic diatheses may not have been passed on to a particular child. One may be a COA without being undercontrolled, having an attention deficit hyperactivity disorder diagnosis, or other problems known to be associated with increased risk of alcohol dependence.

**Risk and Protection**

Although almost all U.S. youth grow up in a culture permeated by alcohol, they are not uniformly at risk for alcohol consumption or its consequences. Much research has addressed the risk and protective factors associated with youth drinking. These factors include but are not limited to family history and genetic vulnerability, comorbid conditions and their developmental antecedents, sociodemographic characteristics, social stressors such as poverty and lack of social support, family characteristics, alcohol availability, temperament, and other individual factors. Some of the most consistently documented epidemiologic findings regarding the association of alcohol consumption and other factors are presented in the following brief overview.

Data from general population surveys of youth, as well as data from smaller, more localized studies, consistently indicate that rates of drinking and alcohol-related problems are highest among White and American Indian or Alaska Native youth, followed by Hispanic youth, African Americans, and Asians. Likewise, studies uniformly indicate that alcohol consumption generally increases as a person’s age increases. Prevalence rates of drinking for boys and girls are similar in the younger age groups; among older adolescents, however, more boys than girls engage in frequent and heavy drinking, and boys show higher rates of drinking problems. Other common findings are a strong association between conduct problems and earlier alcohol consumption, and youth with a family history of alcohol problems are at much greater risk both for problem use and later alcohol use disorders. Studies also show that underage drinkers generally possess more than one risk factor and exhibit clusters of problem behaviors.

The scientific literature on risk and protective factors for underage drinking reveals important conceptual as well as methodological issues. For example, many such risk factors have been identified solely on the basis of their association with drinking and its consequences. This association is not sufficient evidence, however, to prove that something actually increases risk for, or protection from, underage drinking. Some scientists, therefore, advocate a stricter definition of risk/protective factors (e.g., Donovan 2004). The term “risk factor,” for example, would apply only to variables for which there is a statistically significant link to the onset of adolescent alcohol use as well as evidence that any such variable was present prior to the onset of drinking (Donovan 2004). Furthermore, “alcohol consumption” encompasses not just one but numerous phenomena. Finally, it is important to be aware that many of the risk factors predicting early drinking are not drinking variables but instead are more nonspecific characteristics, such as externalizing and internalizing problems, that are identifiable much earlier than the first drinking experience but represent high-risk pathways into earlier use (NIAAA 2000; Zucker and Wong 2005). We therefore need to more precisely define those risk/protective factors that apply to the initiation of drinking, to the escalation of drinking, to risky drinking, and to other aspects of consumption.

ReferencesDonovanJEAdolescent alcohol initiation: A review of psychosocial risk factorsJournal of Adolescent Health35529.e718200410.1016/j.jadohealth.2004.02.00315581536National Institute on Alcohol Abuse and Alcoholism (NIAAA)Alcohol involvement over the life courseTenth Special Report to the US Congress on Alcohol and Health: Highlights From Current ResearchBethesda, MDDept of Health and Human Services, NIAAA20002853Available online at: http://www.niaaa.nih.gov/publications/10report/intro.pdfZuckerRAWongMMPrevention for children of alcoholics and other high risk groupsGalanterMRecent Developments in Alcoholism, Vol. 17: Alcohol Problems in Adolescents and Young Adults: Epidemiology, Neurobiology, Prevention, TreatmentNew YorkSpringer200529932010.1007/0-306-48626-1_1415789872

Socialization risk involves exposure, but given the high divorce rates found in this population, evaluating the level of socialization risk is complex, involving both the quantification of the length of the exposure and the identification of the developmental period during which the socialization took place. Vulnerability is greater during some developmental periods than others (Fuller et al. 2003). In addition, a substantial amount of marital assortment occurs in alcoholic families (Schuckit et al. 2002). When assortment is present, risk exposure is multiplied, and COA effects become a function of genetic risk(s), individual parent risk, and the synergistic risk created by marital interaction (Fuller et al. 2003).

Third, the potential for indirect socialization effects also is higher in COAs than in other children. Parental psychopathology has been documented as a risk factor for poorer parental monitoring (Chilcoat et al. 1996), which in turn leads to a higher probability of involvement with a deviant peer group, including earlier exposure to alcohol and other drugs.

Fourth, COA risk is not simply risk for the development of AUD (Zucker and Wong 2005). Given what is known about the elevated comorbidities found among offspring of alcoholics, this designator also is a marker of elevated risk for behavioral and cognitive deficits. These include attention deficit disorder, behavioral undercontrol/conduct disorder, delinquency, lower IQ, poor school performance, low self-esteem, and other problems (Noll et al. 1992; Nigg et al. 1998; Poon et al. 2000; Sher 1991; West and Prinz 1987). Furthermore, the evidence strongly implicates some of these non-alcohol-specific characteristics as causal to both problem alcohol use and elevated risk for AUD (Caspi et al. 1997; Donovan and Jessor 1985; Nigg et al. 1998).

These factors implicate the COA population as an important component of the underage drinking population. For the same reasons, however, it is essential to determine which components of that risk composite are the strongest mediators of the underage drinking outcome.

### College Students

College students are a highly visible group of underage drinkers among whom alcohol consumption is commonplace. Indeed, many college students accept alcohol use as a normal part of student life. Studies consistently indicate that about four in five college students drink alcohol; about two in five engage in episodic heavy consumption, often called bingeing (five or more drinks in a row for men and four or more in a row for women; generally asked with respect to the past 2 weeks or past 30 days, depending on the survey); and about one in five engages in frequent episodic heavy consumption (bingeing three or more times in the past 2 weeks) (NIAAA 2002).

The consequences of drinking among college students include academic problems, social problems, legal problems, involvement in physical and/or sexual assault or risky sex, and even death. An estimated 1,700 college students between the ages of 18 and 24 die each year from alcohol-related unintentional injuries including motor vehicle crashes (Hingson et al. 2005). Another 599,000 students are unintentionally injured while under the influence of alcohol, 696,000 are assaulted by other students who have been drinking, and 97,000 are victims of alcohol-related sexual assault or date rape (Hingson et al. 2005). A striking number of college students also report having experienced alcohol-induced memory blackouts. One recent study indicated that among nonabstaining college students, 40 percent reported experiencing a blackout within the past year, and 9.4 percent reported having a blackout within the past 2 weeks (White et al. 2002). This could relate to students’ tendency to be unaware of standard drink volumes and to overpour drinks, thus underestimating their consumption (White et al. 2003). It is not known if younger drinkers are more susceptible to the memory-impairing effects of alcohol, but one study in humans showed that a dose of alcohol resulting in a BAC in the range of 80 mg/dl significantly disrupted learning in people in their early twenties but had little effect on people in their late twenties (Acheson et al. 1998).

Drinking in college varies from campus to campus and from person to person. Levels and patterns of consumption are associated with individual, intracampus, and intercampus factors. For example, athletes and members of fraternities and sororities are among the heaviest drinkers on most campuses, and students in the Northeast and on campuses where athletics and Greek organizations are prominent tend to drink more than their counterparts at other institutions (NIAAA 2002).

### Underage and Youthful Drinking Among Military Personnel

The Department of Defense (DOD) conducts periodic surveys to assess alcohol use and other health-related behaviors among military personnel. Approximately 193,000 of 1.4 million active duty military personnel are between the ages of 17 and 20. These surveys, therefore, provide important information about underage drinking in an important subset of young people. According to the 2002 DOD survey (the most recent one for which results have been released):

- 33.3 percent of military personnel age 20 and younger are “abstainers” (drink once a year or less).
- 15.7 percent are “infrequent/light” drinkers (one to four drinks per typical occasion, one to three times per month).
- 10.4 percent are “moderate” drinkers (one drink per typical drinking occasion at least once a week, *or* two to four drinks per typical drinking occasion two to three times per month, *or* five or more drinks per typical drinking occasion once a month or less).
- 14.4 percent are “moderate/heavy” drinkers (two to four drinks per typical drinking occasion at least once a week *or* five or more drinks per typical drinking occasion two to three times per month).
- 26.1 percent are “heavy” drinkers (five or more drinks per typical drinking occasion at least once a week).

A comparison of data from the 2002 DOD survey with data from the 2001 NHSDA (in which heavy alcohol use is defined as five or more drinks on one occasion on 5 or more days in the past 30 days) indicates that rates of heavy drinking among 18- to 25-year-olds in the military are higher than for civilians of the same age (32.2 percent vs. 17.8 percent for men and 8.1 percent vs. 5.5 percent for women).

The surveys conducted by the DOD also indicate that substantial numbers of youth in the military experience negative consequences from drinking. The 2002 data show that, during the 12 months prior to the survey, more than one-fifth of the most junior enlisted personnel (who typically are between the ages of 17 and 20) experienced serious consequences as a result of drinking or a drinking-related illness, including military punishment, alcohol-related arrest, and the need for detoxification, and that more than one-fourth experienced a productivity loss because of alcohol use. DOD investigators classified more than one-fifth of survey participants as alcohol “dependent” based on the number of days during the previous 12 months that they reported (1) withdrawal symptoms, (2) inability to recall things that happened while drinking, (3) inability to stop drinking before becoming drunk, or (4) morning drinking.

### Minority Youth

According to national surveys, there is considerable variation between Whites and ethnic/racial minorities with respect to alcohol consumption. Minority youth generally start drinking at older ages than their White non-Hispanic counterparts. A greater difference also exists in levels of drinking between male and female minority youth and a greater percentage of minority youth abstain or drink very little (Vega et al. 1993). Although a “typical” pattern of underage drinking could never be attributed to any specific minority group, it is useful to compare minority groups to identify potential risk and protective factors that may be operating to produce some of the observed differences in drinking practices. With a burgeoning minority population, it is also essential to better understand these factors to help design and implement the most effective prevention and intervention programs.

Data from a recent nationwide survey reveal that about three-fourths of White, American Indian, and Hispanic high school seniors used alcohol in the past year. More than 6 percent of American Indian and 5 percent of Mexican and Cuban American seniors report daily drinking, compared with 1 percent to 3.8 percent for all other groups (Wallace et al. 2002). The limited data on Hispanic and American Indian adults suggest that, among those who drink, there is a tendency toward high average intake per drinking day. Youth survey data suggest that some students establish a pattern of heavy drinking by their senior year of high school. About 60 percent of African American and about 57 percent of Asian American high school seniors report having used alcohol in the past 12 months, and about 32.5 percent in both groups report having used it in the past 30 days. However, there appears to be a “cross-over” effect for African Americans. That is, even though they use less alcohol as youths than their non-Hispanic White counterparts, rates of heavy and problem drinking among African American adults, especially males, are higher than for non-Hispanic Whites.

Studies of race and ethnicity should be conducted with sufficiently large and diverse samples to allow investigators to assess variations in drinking by national origin or tribal affiliation, acculturation, immigration status, and language. Significant variation exists among Hispanics and among American Indians. Recent evidence indicates that members of some American Indian groups are more likely to abstain than are people in the general U.S. population. Like their Mexican American counterparts, American Indian drinkers, however, consume more alcohol per drinking occasion (Beals et al. 2003). In addition, although Asian Americans often are considered the “model minority,” with low rates of alcohol use, most current literature does not include data from rapidly growing at-risk Asian groups such as Southeast Asians, Koreans, and Filipinos, or groups believed to have higher rates of alcohol use, such as Native Hawaiians and other Pacific Islanders (Zane and Sasao 1992).

Although there is clear evidence of genetic variability in alcohol metabolism, we have yet to fully understand the interplay of genetic and environmental variables. For example, the inability to metabolize alcohol efficiently, deemed a protective factor in a subset of the Asian population because of the unpleasant effects of drinking, often results in facial flushing. Highlighting the complexity of the interplay between genetic and environmental variables is the observation that Asian American drinking often increases with level of acculturation in spite of the flushing response (Sue et al. 1979).

## Figures and Tables

**Figure 1:**
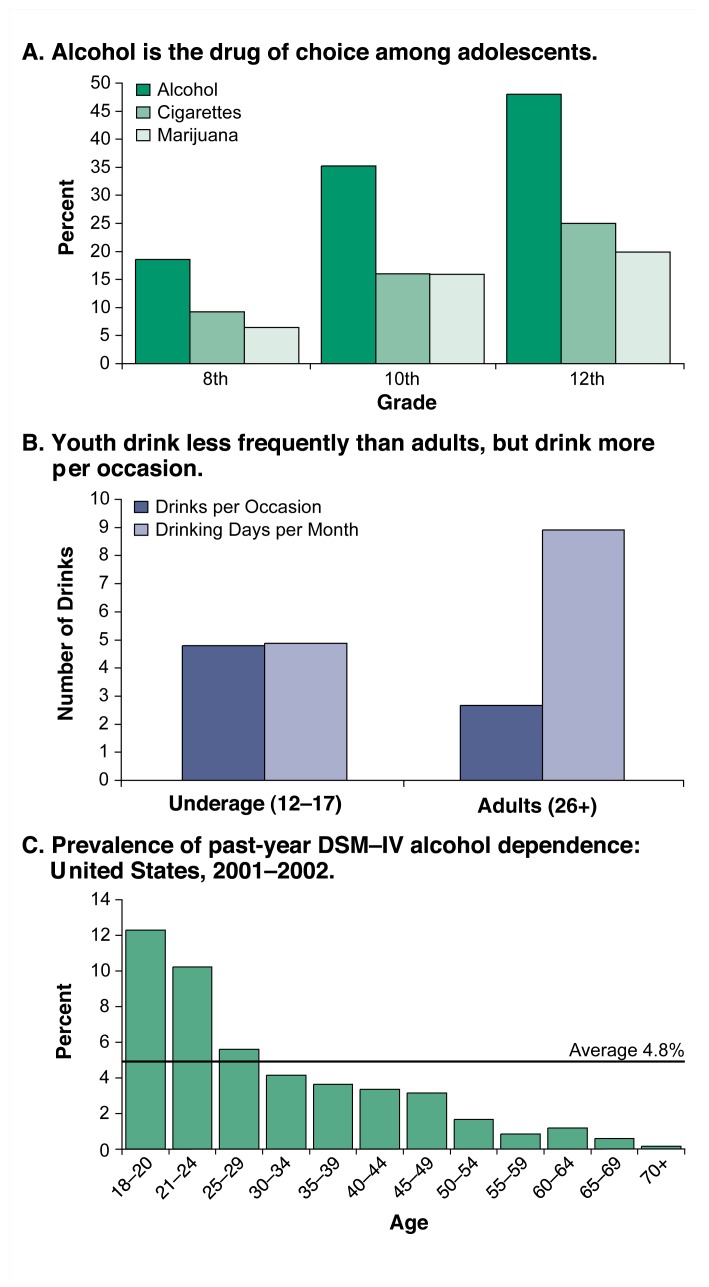
Nationwide surveys, as well as studies in smaller populations, show that drinking is widespread among people under age 21. SOURCES: A. SAMHSA 2003; Johnston et al. 2005; B. SAMHSA 2003; Johnston et al. 2003; C. NIAAA 1998.

**Figure 2:**
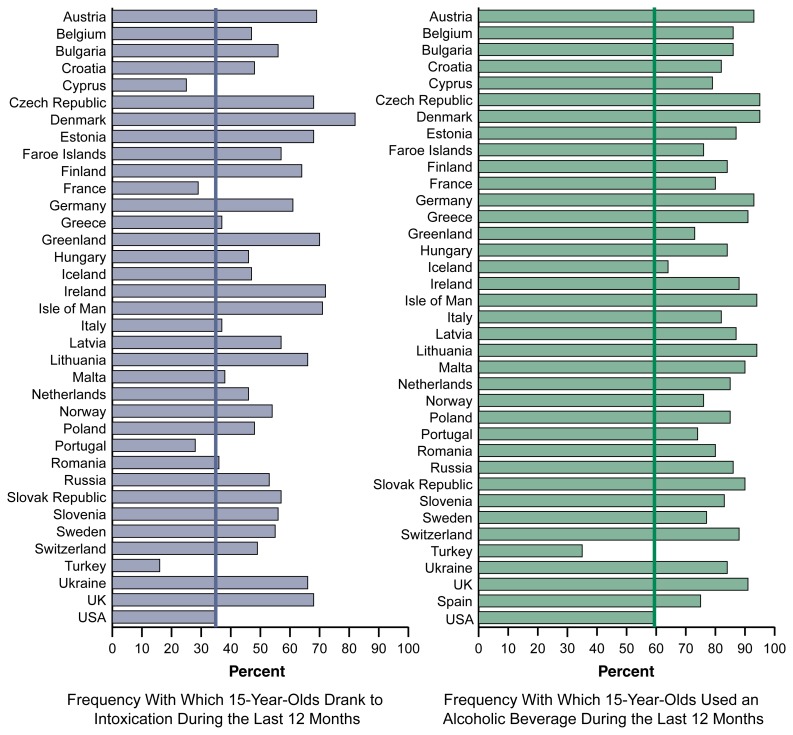
Alcohol use by youth is an international phenomenon. The 2003 European School Survey Project on Alcohol and Other Drugs (ESPAD) surveyed 15-year-olds in 35 European countries where legal drinking ages are lower (typically ages 16–18) than in the United States. The ESPAD questions were similar to those used with 10th graders in the U.S. Monitoring the Future study. In all European countries except the predominantly Moslem nation of Turkey, a greater percentage of 15-year-olds drank alcohol than in the United States; and in more than three-quarters of the countries, a greater percentage reported drinking to intoxication in the previous year than in the United States. SOURCE: http://www.espad.org/reports.html

## Footnotes

1 SAMHSA’s definitions of binge and heavy drinking: binge drinkers report that they had consumed five or more drinks on the same occasion at least once in the past 30 days; heavy drinkers report that they had consumed five or more drinks on the same occasion on at least 5 different days in the past 30 days (SAMHSA 2003).
